# Cutaneous Epstein-Barr virus-positive polymorphous posttransplantation lymphoproliferative disorder responding to rituximab

**DOI:** 10.1016/j.jdcr.2025.07.015

**Published:** 2025-08-16

**Authors:** Maggie Chen, Jacqueline Hwang, Juris Germanas

**Affiliations:** a Department of Dermatology, University of Maryland Medical Center, Baltimore, Maryland; b Department of Dermatology, Veterans Affairs Medical Center, Baltimore, Maryland

**Keywords:** Epstein-Barr virus (EBV) infections, immunosuppressive therapy, lymphoproliferative disorders, organ transplantation

## Introduction

Posttransplant lymphoproliferative disorders (PTLDs) are a group of rare but serious conditions that result in excess production of lymphoid cells after solid organ or bone marrow transplantation. PTLD is a major contributor to cancer-related deaths and graft loss, and, in contrast to other non-Hodgkin lymphomas, occurs frequently within extranodal sites, such as the gastrointestinal tract and central nervous system.^1^ The majority of cases are associated with Epstein-Barr virus (EBV) and develop most commonly within the first year after transplantation. Here we report a rare case of cutaneous polymorphic PTLD in a kidney transplant patient that occurred more than 10 years after allograft introduction, and its response to treatment with rituximab.

## Case report

**Fig 1:**
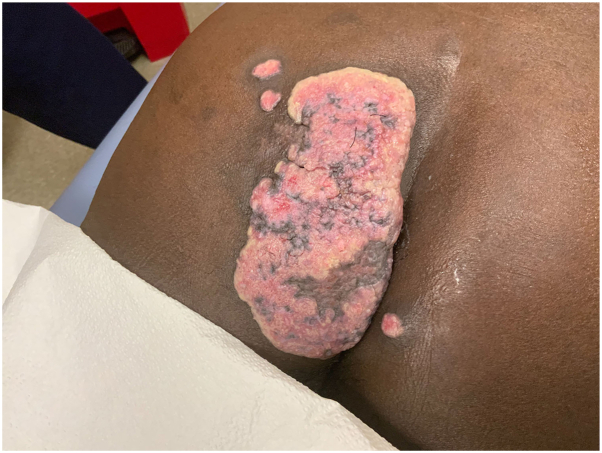
Posttransplant lymphoproliferative disorder. Initial presentation of a 12 cm x 6 cm beefy-red, sclerotic, well-demarcated plaque on the left buttock.

A 60-year-old man with past medical history of kidney transplant on chronic immunosuppression for over 10 years with tacrolimus 0.5 mg twice a day, prednisone 2.5 mg daily, and mycophenolate 1000 mg twice a day presented with a 12 cm × 6 cm beefy-red, sclerotic, well-demarcated plaque on his left buttock (Fig 1). Per the patient’s report, the lesion first appeared as a small red papule, but over 4 months, rapidly enlarged with continuous serous drainage. He denied any cough, flu-like symptoms, weight loss, or recent travel in the past 5 years. Notably, he also had a history of coccidiomycosis managed with fluconazole 200 mg daily for long-term suppression therapy.

**Fig 2:**
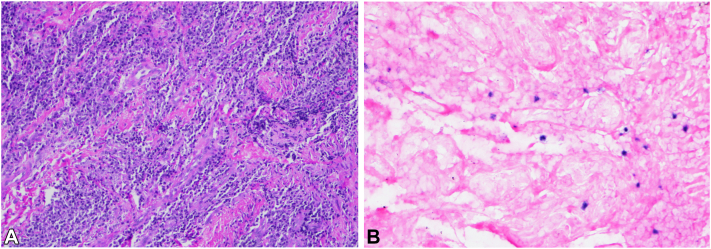
Posttransplant lymphoproliferative disorder. **A,** Histopathologic examination of specimen from left buttock reveals dermis infiltrated by small lymphocytes and plasma cells in the background of collagen fibrosis. **B,** Epstein-Barr encoding region stain demonstrating foci of Epstein-Barr virus-positive cells.

A punch biopsy obtained showed infiltration of the dermis by lymphocytes and plasma cells in a background of collagen fibrosis (Fig 2, *A*). Immunohistochemistry stains were positive for CD79, CD30, Mum1, and CD138. Reactive background T lymphocytes were positive for CD3 and CD5. In situ hybridization for Epstein-Barr encoding region was also positive and showed a focus of EBV^+^ cells (Fig 2, *B*). Special stains for acid-fast bacteria and periodic acid–Schiff with fungal stain with appropriate controls were negative. Negative markers included CD23, CD21, CD56, and CD117. Kappa and lambda immunostains demonstrated plasma cells with a polytypic pattern of reactivity. Ki67 demonstrated some foci with a proliferation index of approximately 20%. Collectively, the findings were consistent with polymorphic PTLD.

**Fig 3:**
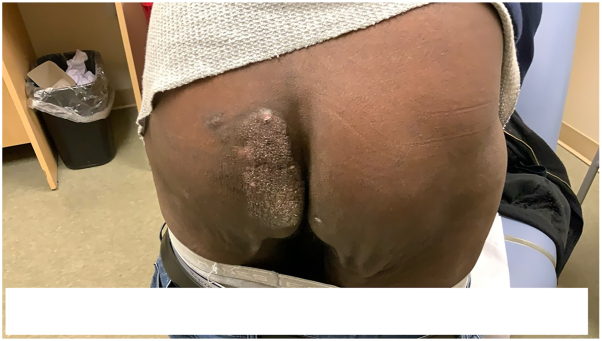
Posttransplant lymphoproliferative disorder. Clinical appearance of lesion after reduction of immunosuppression and 8 infusions of rituximab.

After evaluation by oncology and nephrology, the patient was tapered off prednisone and mycophenolate mofetil, but continued on tacrolimus 1.0 mg twice a day (twice his initial dose). Rituximab monotherapy (375 mg/m^2^) weekly was then initiated, and the patient received 8 infusions over a 12-week period. At his follow-up appointment, the patient demonstrated substantial reduction in size of the buttock lesion (Fig 3), consistent with strong clinical response to therapy. Further positron emission tomography-computed tomography demonstrated significant reduction in fluorodeoxyglucose activity within the lesion (maximum standardize uptake value decreased to 2.4 from 4.9 before therapy). Currently he is off therapy and has been free of recurrence for over 6 months.

## Discussion

PTLD are a group of rare but serious conditions that result in excess production of lymphoid cells after solid organ or bone marrow transplantation.^1^ PTLD is a major contributor to cancer-related deaths and graft loss, and often manifests in areas outside of the lymph nodes. The majority of cases are associated with EBV. Notably, EBV specifically can promote B-cell transformation in the setting of impaired T-cell function, which increases the risk of malignant transformation. Pretransplant EBV seronegativity, in fact, increases the risk of developing the condition by up to 75 times.^2^

Other risk factors for developing PTLD include patient age, immunosuppressive regimen pursued, and transplant organ type.^3^ Multiorgan recipients face the highest risk, whereas kidney transplant recipients have the lowest. Diagnosis relies on pathology, with common findings such as polymorphous infiltrate and atypical large B-cell blasts coexpressing B-cell antigens and CD30, often with Hodgkin/Reed Sternberg cell-like morphology.^1^ Dependent on histopathologic findings, PTLD is classified into nondestructive, polymorphic, monomorphic, and Hodgkin lymphoma-like types.^1^

Primary cutaneous PTLD is uncommon, comprising only 5% of PTLD cases.^4^, ^5^, ^6^, ^7^, ^8^ Clinically, cutaneous PTLD can present as erythematous nodules or plaques on the skin, or painful, sharply circumscribed mucocutaneous ulcers. General categorizations include B or T-cell subtypes, with polymorphic variants of cutaneous PTLD being considered rare variants. A summary of reported cases of cutaneous PTLD, along with salient features and outcomes of treatment is seen in Table I.^4^, ^5^, ^6^, ^7^, ^8^

**Table I:** Features of previously reported cases of cutaneous PTLD

Reference	Patient age and gender	Type of PTLD	Treatment	Outcome
Capaldi et al^6^	69-year-old woman	EBV^+^ polymorphic primary cutaneous PTLD	Cyclosporine, prednisone, and acyclovir	Within 2 mo the lesions resolved. Died from end-stage renal disease 2 y later
Salama et al^5^	Case 1: 73-year-old woman	EBV^+^ large B-cell lymphoma	Cyclosporine, prednisone, and palliative CHOP	Died from sepsis
	Case 2: 54-year-old man	EBV^+^ large B-cell lymphoma	Cyclosporine, prednisone, and acyclovir	Died 1.5 y later from unrelated medical causes
	Case 3: 64-year-old man	Plasmacytoma	Radiation, chlorambucil, acyclovir, cyclosporine, prednisone, and mycophenolate mofetil	Died 8 y later from unrelated medical causes
	Case 4: 59-year-old man	EBV^−^ CD30^+^ anaplastic large T-cell lymphoma	Radiation, chlorambucil, cyclosporine, prednisone, and mycophenolate mofetil	Died from heart failure 2 y later
Samolitis et al^4^	63-year-old man	EBV^+^ plasmacytoid tumor	Cyclosporine and prednisone	Cutaneous tumors resolved with therapy, and did not recur within the 9-mo of follow-up Died from cardiac failure
Beynet et al^8^	Case 1: 60-year-old man	EBV^+^, large B-cell lymphoma	Cyclosporine, acyclovir, and rituximab therapy (375 mg/wk for 4 wk)	All lesions resolved by 4 wk. At the 1.5-y follow-up, patient remained alive without recurrence
	Case 2: 49-year-old woman	Polymorphic PTLD (insufficient tissue for EBV analysis)	Cyclosporine, prednisone, and localized radiation therapy	At the 2-y follow-up, patient remained alive without recurrence
	Case 3: 71-year-old man	EBV^+^, polymorphic PTLD	Cyclosporine, prednisone, and acyclovir	At the 5-y follow-up, patient remained alive without recurrence
	Case 4: 13-year-old woman	EBV^+^, monomorphic PTLD	Cyclosporine, ganciclovir, and rituximab (400 mg/wk for 3 wk)	At the 2-y follow-up, patient remained alive without recurrence
Schumann et al^7^	61-year-old woman	EBV^+^, polymorphic lymphoma	Cyclosporine, azathioprine, prednisone, and acyclovir	Died from sepsis

*CHOP*, Cyclophosphamide, doxorubicin, vincristine, and prednisone; *EBV*, Epstein-Barr virus; *PTLD*, posttransplant lymphoproliferative disorder.

Treatment of PTLD depends on disease severity. First-line treatment involves a reduction of immunosuppression while preserving the transplanted organ.^9^ However, additional therapeutic interventions are frequently needed when there is a lack of clinical improvement. Primary cutaneous PTLD may be treated by surgical excision and local radiation therapy for isolated lesions.^3^ Progressive PTLD may benefit from combination chemotherapy regimen including cyclophosphamide, doxorubicin, vincristine, and prednisone, although limitations may include hematotoxicity, organ dysfunction, and infection. Systemic treatment options such as rituximab, a chimeric anti-CD20 monoclonal antibody, can be useful for B-cell PTLD and there is growing evidence demonstrating benefit as a first-line therapy.^9^

## Conflicts of interest

None disclosed.
